# A Survey of Intestinal Helminths of Dogs in Slovakia with an Emphasis on Zoonotic Species

**DOI:** 10.3390/ani11103000

**Published:** 2021-10-19

**Authors:** Júlia Jarošová, Daniela Antolová, Branislav Lukáč, Aladár Maďari

**Affiliations:** 1 Institute of Parasitology, Slovak Academy of Sciences, Hlinkova 3, 040 01 Košice, Slovakia; antolova@saske.sk; 2 Small Animal Clinic, University of Veterinary Medicine and Pharmacy in Košice, Komenského 73, 041 81 Košice, Slovakia; branislav.lukac@uvlf.sk (B.L.); aladar.madari@uvlf.sk (A.M.)

**Keywords:** dogs, zoonotic parasites, *Toxocara* spp., *Ancylostoma*/*Uncinaria* spp., *Echinococcus multilocularis*

## Abstract

**Simple Summary:**

Dogs are the most popular pet animals worldwide; however, frequent and close contact with people increases the risk of transmission of different zoonotic parasites. As the occurrence of intestinal parasites in the dog population is affected by several factors, understanding the epidemiology of zoonotic parasitic infections is important to minimize the risks for humans. This study presents results about the prevalence of gastrointestinal helminths in seven different groups of dogs (pet, shelter, guard, working, and hunting dogs, as well as dogs from segregated Roma settlements) in Slovakia. Out of 495 faecal samples collected between 2016 and 2021, eggs of intestinal helminths were detected in 134 (27.1%) samples. Altogether, six different species/genera/families, namely, *Toxocara canis* (14.7%), *Toxascaris leonina* (1.6%), *Trichuris* *vulpis* (6.3%), *Capillaria* spp. (1.4%), *Ancylostoma*/*Uncinaria* spp. (8.3%), and taeniid eggs (4.0%), were recorded. Infection with *Echinococcus multilocularis* was confirmed in 2.2% of dogs and 0.4% of the animals were infested with *Taenia hydatigena*. The results showed that the occurrence of intestinal helminths is quite frequent in the majority of analyzed dog groups, with a close correlation between the occurrence of intestinal helminths and availability of veterinary care and anthelmintic therapy.

**Abstract:**

Dogs are the most popular pets worldwide; however, close contact with people increases the risk of transmission of different zoonotic parasites. This study aims to determine the prevalence of gastrointestinal helminths in dogs in Slovakia. A total of 495 faecal samples collected from pet, shelter, guard, working (police), and hunting dogs, as well as dogs from segregated Roma settlements between 2016 and 2021, were examined using flotation and molecular methods. Eggs of intestinal helminths were detected in 134 (27.1%) samples. Microscopically, six different species/genera/families, namely, *Toxocara canis* (14.7%), *Toxascaris leonina* (1.6%), *Trichuris* *vulpis* (6.3%), *Capillaria* spp. (1.4%), *Ancylostoma*/*Uncinaria* spp. (8.3%), and taeniid eggs (4.0%), were recorded. Molecular analyses revealed infection with *Echinococcus multilocularis* in 2.2% of dogs and 0.4% of the animals were infected with *Taenia hydatigena*. The results showed a correlation between the occurrence of intestinal helminths and the availability of veterinary care, as dogs from Roma settlements and shelter dogs were the most often infected (66.7% and 39.2%, respectively). On the other hand, working animals were in the best health condition, with only 2.5% being positive. The relatively frequent occurrence of zoonotic species points to the constant need for preventive measures and regular deworming of dogs.

## 1. Introduction

Dogs are the most popular pets worldwide, and except for many direct or material benefits when used, e.g., as guard, shepherd, hunting, or therapeutic dogs, they also provide their owners many positive psychological benefits [1]. However, close contact between dogs and people also increases the risk of transmission of different zoonotic diseases [2,3].

The number of dogs living in close proximity to humans can contribute to a high rate of soil and grass contamination with infective parasitic stages in leisure, recreational, public, and urban areas [4]. Parasitic elements, like eggs, larvae, and oocysts excreted via canine faeces, can survive over a long time and remain infective in the environment under different conditions. Therefore, the environmental faecal contamination of public areas is a global health problem that is difficult to control [5,6,7]. Several endoparasites of dogs such as *Toxocara canis*, *Ancylostoma caninum*, *Dipylidium caninum,* and *Echinococcus* spp., as well as some other taeniid species and *Uncinaria* spp., can cause infections in humans [8,9].

In recent decades, the occurrence of intestinal parasites in the dog population has been affected by several factors. As canned dog food cannot be a source of double-host parasites, these species have gradually disappeared from the dog population [10]. On the other hand, the risk of transmitting parasitic diseases increases with the more frequent contact of dogs with free-living carnivores or with the possibility of catching rodents. Therefore, hunting dogs, stray dogs, or dogs that live outside may face a higher risk of being infected [11,12]. Moreover, the effort to return to the former/original form of the dog diet has appeared recently and a growing number of dog owners have started to feed their dogs with raw meat, meat products, and bones, the so-called raw meat-based diet [13,14]. Thus, the risk of transmission of parasitic species that can be spread via raw meat has increased again.

Understanding the epidemiology of zoonotic parasitic infections is important for minimizing the risks for humans [15]. Therefore, the objective of the present study was to determine the prevalence of gastrointestinal helminths in dogs in Slovakia.

## 2. Materials and Methods

A total of 495 dog faecal samples were collected from different localities in Slovakia between 2016 and 2021. The samples were obtained from pet dogs (*n* = 194), shelter dogs (*n* = 97), dogs from segregated Roma settlements (*n* = 45), hunting dogs (*n* = 67), guard dogs (*n* = 13), and working dogs (*n* = 79).

The category “pet dogs” encompassed animals kept in households for companionship or other animals kept under the supervision of the owner, with restricted and controlled movement in the countryside. The dogs were not dewormed at least three months before sampling. The group of “shelter dogs” comprised stray, lost, abandoned, or surrendered animals that had been caught and put into shelters. Faecal samples from shelter dogs were taken before deworming after they came to the shelters. Animals assigned to “dogs from segregated Roma settlements” were usually kept under poor hygienic conditions without proper veterinary control and usually roaming freely all over the settlements and their surroundings. The category of “hunting dogs” included animals kept by hunters. Animals assisted hunters in finding, pursuing, and retrieving game during hunting. These dogs could be in contact with free-living carnivores and are often allowed to catch rodents. The category “working dogs” encompassed mostly animals specifically trained to assist police (i.e., police dogs). “Guard dogs” were kept to protect property, mostly in companies or factories. These animals were kept under the supervision of handlers, usually with controlled movement over a fenced-in area. The dogs were not dewormed at least three months before sampling.

After collection, the faecal samples were transported to the laboratory where they were stored at +4 °C before analysis. The parasitological examination was performed within 48 hours. Samples were investigated for the presence of propagative stages of endoparasites using a modified Faust’s flotation method [16]. All eggs found were identified according to their morphological characteristics under light microscopy. As the eggs of *Ancylostoma caninum* and *Uncinaria stenocephala* are very similar and hardly distinguishable by microscopy, hookworm eggs found in the study are reported as *Ancylostoma*/*Uncinaria* spp. eggs.

### 2.1. Molecular Analyses

Twenty faecal samples positive for taeniid eggs were analysed by PCR-derived methods. To disrupt the parasite eggshells, faecal samples were homogenized in a Qiagen TissueLyser 85210 (Retsch, Haan, Germany) using 5 mm stainless steel beads for 6 min (30 Hz). After this step, the genomic DNA was isolated using the QIAamp DNA Stool Mini Kit (Qiagen, Hilden, Germany) according to the manufacturer’s instructions. PCR reactions were performed using the 5× FIREPol^®^ Master Mix Ready to Load (SOLIS Biodyne, Tartu, Estonia). For detection of *Echinococcus* spp. tapeworms, a nested PCR reaction was used. Amplification of the partial 12S rRNA gene was performed using specific primers; for the first step primers, P60-for and P375-rev designed by Dinkel et al. [17] were used. To detect *E. multilocularis*, the second step was performed with the Em-nest-for and Em-nest-rev primers designed by Dyachenko et al. [18]. For *E. granulosus sensu stricto* (*s.s.*), the primer pair E.g.ss1for and E.g.ss1rev, and for *E. canadensis*, the primers E.g.cs1for and E.g.cs1rev were used [19]. To detect other taeniid species, amplification of a 471 bp region of the *nad1* gene was applied with the JB3 and JB4.5 primer set, as described by Bowles and McManus [20].

### 2.2. Statistical Analyses

The prevalence values of parasitic infection in dogs were provided with a 95% confidence interval (95% CI). The Chi-squared (χ2) test was used to test the differences among the prevalence of parasitic species and the occurrences of parasites in dog categories, with a value of *p* < 0.05 considered significant. The statistical analyses were performed using the Quantitative Parasitology on the Web software [21].

## 3. Results

Out of 495 dog faecal samples, the presence of the propagative stages of parasites was detected in 134 samples, representing an overall prevalence of 27.1%. Microscopically, six different species/genera/families of intestinal helminths were detected in the examined animals, namely, *Toxocara canis* (14.7%), *Toxascaris leonina* (1.6%), *Trichuris vulpis* (6.3%), *Capillaria* spp. (1.4%), *Ancylostoma*/*Uncinaria* spp. (8.3%), and taeniid eggs (4.0%) (Table 1).

Molecular analyses of samples positive to taeniid eggs revealed infection with *Echinococcus multilocularis* in 11 (2.2%) dogs and 2 animals (0.4%) were infected with *Taenia hydatigena*. None (0.0%) of the animals were infected with *E. granulosus s.s.* or *E. canadensis.*

Parasitic species with zoonotic potential, namely, *T. canis*, *Ancylostoma*/*Uncinaria* spp., and *E. multilocularis,* were identified in 22.0% of dog faeces.

The prevalence of *T. canis* was significantly higher (*p* < 0.05) than the prevalence of other parasites. A significantly higher positivity was also recorded when comparing the occurrence of *Ancylostoma/Uncinaria* spp. with *T. leonina*, *Capillaria* spp., *Echinococcus* spp., and taeniid species prevalence.

A total of 102/495 (20.6%) dogs were infected by only one parasite species. Mixed infections caused by two or three parasitic species were discovered in 4.6% (23/495) and 1.8% (9/495) of animals, respectively (Table 2). The most frequent was the *T. canis*/*T. vulpis* and *Ancylostoma*/*Uncinaria* spp./*T. vulpis* combination that occurred in six and five dogs, respectively. Mixed infections caused by three helminth species was most often (in four dogs) caused by *T. canis,* *Ancylostoma*/*Uncinaria* spp., and *T. vulpis.*

Intestinal parasites were most commonly detected in dogs from segregated Roma settlements in which the prevalence (66.7%) was significantly higher (*p* < 0.05) than in all the other groups (pet, shelter, guard, working, and hunting dogs). The most frequently observed parasite in this group was *Ancylostoma*/*Uncinaria* spp. (35.5%), followed by *T. canis* (31.1%), *Taenia* spp. (13.3%), and *T. vulpis* (13.3%). The most frequent occurrence of mixed infections was also recorded in this category (11/45), with *T. canis/Ancylostoma*/*Uncinaria* spp. being the most common combination.

In the population of shelter dogs, six parasitic species—*T. canis* (27.8%), *T. leonina* (1.0%), *T.* *vulpis* (9.3%) *Ancylostoma*/*Uncinaria* spp. (15.5%), *E. multilocularis* (1.0%), and *Taenia* spp. (1.0%)—were detected. Shelter dogs were infected more often than pet and working dogs (*p* < 0.05), but there were no significant correlations with guard or hunting animals.

The most parasitic species were identified in “pet dogs”, namely, *T. canis*, *T. leonina*, *T. vulpis*, *Ancylostoma*/*Uncinaria* spp., *Capillaria* spp., *E. multilocularis*, *T. hydatigena,* and *Taenia* spp., with a total of 46 (23.7%) positive animals.

The overall positivity of guard and hunting dogs was similar, reaching 23.1% and 22.4%, respectively.

Working dogs were less frequently infected (*p* < 0.05) than the dogs of all other categories. One dog (1.3%) was infected with *Toxocara canis* and one (1.3%) with *Ancylostoma/Uncinaria* spp. (Table 1).

## 4. Discussion

Most of the dog intestinal helminths identified in the present study are cosmopolitan in their distribution, but the prevalence of each species was affected by the conditions under which the animals were kept. The overall prevalence of intestinal endoparasites was 27.1%, revealing a relatively frequent occurrence of parasitic infections. *Toxocara canis*, *Ancylostoma*/*Uncinaria* spp., and *Trichuris* *vulpis* were the most prevalent species, reaching 14.7%, 8.3%, and 6.3% prevalence, respectively. Altogether, 22.0% of dogs were infested with zoonotic species, namely, *T. canis*, *Ancylostoma*/*Uncinaria* spp., and *Echinococcus multilocularis.* Human *Toxocara* infection occurs after the accidental ingestion of embryonated eggs from the environment or larvae from undercooked tissues of infected paratenic hosts. Toxocariasis manifests in a range of clinical syndromes, which include visceral and ocular larva migrans, neurotoxocariasis, and covert toxocariasis [22]. *A. caninum* has been reported to have the potential to cause eosinophilic enteritis, cutaneous larva migrans, or neuroretinitis [23,24]. Similarly, *U. stenocephala* can cause cutaneous larva migrans in humans [25,26,27]. Although in Slovakia, human cases of cutaneous larva migrans have so far been reported mostly as imported [28,29,30], the 8.3% overall prevalence of *Ancylostoma*/*Uncinaria* spp. recorded in our study highlights the risk of autochthonous infection.

The less frequent—however, regarding human health, the most dangerous—zoonotic parasite recorded in this study was *E. multilocularis* (2.2%). It causes alveolar echinococcosis, a severe human infection that arises after the accidental ingestion of infective eggs from the contaminated environment, and leads to serious health problems connected primarily with metacestode proliferation in the liver. Without careful clinical management, the disease has a poor prognosis and can result in the death of the patient [31,32]. The non-detection of *Dipylidium caninum* is likely to be related to the poor sensitivity of coproscopy for the detection of this parasite species. Taking into account also the capability of proglotids of moving several inches per hour, the presence/absence of *D. caninum* is not considered to be valid in this survey.

In terms of the use of dogs, the most commonly infected were dogs from segregated Roma settlements, where the overall prevalence of parasitic infections reached 66.7%. In this group, seven different species of parasites—*T. canis*, *T. leonina*, *T. vulpis*, *Capillaria* spp., *Ancylostoma*/*Uncinaria* spp., *E. multilocularis*, and *Taenia* spp.—were detected. In these dogs, mixed infections were also most common. The high prevalence of parasites observed in this population may be easily explained, as these animals do not undergo any health control measures and often starve, and thus catch rodents or feed on garbage, which presents a frequent supplementation source to their diet. In dogs from segregated Roma settlements, the most frequently observed parasite was *Ancylostoma*/*Uncinaria* spp., with 35.5% positivity. The second most prevalent species was *T. canis* (31.1%). A dog infected with adult worms of *T. canis* eliminates thousands of eggs each day [33]. In Roma settlements, a large number of people live together with domestic animals. Within the vicinity of such settlements, animal excrements and human faeces concentrate without any appropriate sanitary control [34]. Therefore, the high prevalence of *T. canis* represents a significant risk for the circulation of infection among animals and its spread to people. A significantly higher risk of human *Toxocara* infection in segregated Roma settlements was confirmed in the study of Antolová et al. [35], who recorded 22.1% seropositivity to *Toxocara* in 429 Roma inhabitants of segregated settlements, while only 4 (1.0%) out of 394 samples derived from the non-Roma population were found to be positive. A similar trend was also recorded in Roma children, in whom 40.3% seropositivity (29/67) was recorded, in contrast to only one positive child (2.3%) from the non-Roma population [36].

Shelter dogs were also commonly infected, with parasite eggs observed in 39.2% of faecal samples. As these dogs usually do not receive attention from their owner or do not even have an owner, and in most cases, rarely or never receive antiparasitic treatment, they are at a higher risk of being infected by intestinal parasites. In this category, the most prevalent and also zoonotic parasites were *T. canis* (27.8%) and *Ancylostoma*/*Uncinaria* spp. (15.5%), and *E. multilocularis* was also detected in one (1.0%) animal. As dogs in shelters are often free-ranging before arriving at the facility, environmental contamination with parasite eggs has likely already occurred over a fairly dispersed area, resulting in the presence of infectious stages that also pose a risk of infection to owned dogs [37]. A similar prevalence of *T. canis* (28.1%) was reported in Slovakia in dogs from shelters in the study of Szabová et al. [11], but the occurrence of *Ancylostoma*/*Uncinaria* spp. eggs was higher (26.8%) at that time.

In our study, the overall prevalence of intestinal parasites in pet dogs was 23.2%. In this group of dogs, seven parasitic species, namely, *T. canis* (11.3%), *T. leonina* (2.1%), *T.* *vulpis* (5.7%), *Capillaria* spp. (2.1%), *T. hydatigena* (1.0%), *E. multilocularis* (2.6%), and *Taenia* spp. (1.5%), were detected. The relatively frequent occurrence of parasites in pet dogs may be related to the fact that, especially in villages, these animals are often fed raw meat and have the opportunity to catch rodents. Nonetheless, many pet dogs do not receive consistent veterinary care.

The lower prevalence of parasites in guard and working dogs may be related to the controlled movement of the animals. Moreover, police (working) dogs are predominantly fed with commercial dog food, are regularly dewormed, and are under veterinary control.

## 5. Conclusions

The results of the presented study showed that the occurrence of intestinal helminths is quite frequent in the majority of the analysed dog groups. A close correlation between the availability of veterinary care and anthelmintic therapy was recorded, as dogs from Roma settlements and shelter dogs were positive the most often. On the other hand, working (police) animals were in the best health conditions in terms of helminthic infections. The relatively frequent occurrence of zoonotic species points to the constant need for preventive measures and regular deworming of dogs.

## Figures and Tables

**Table 1:** Occurrence of intestinal helminths in dogs based on their origin.

Helminth Species	Pet Dogs *n* = 194 (N/%)	Shelter Dogs *n* = 97 (N/%)	Dogs from Segregated Roma Settlements *n* = 45 (N/%)	Guard Dogs *n* = 13 (N/%)	Working Dogs *n* = 79 (N/%)	Hunting Dogs *n* = 67 (N/%)	Total	
							*n* = 495 (N/%)	95% CI
Toxocara canis *	22/11.3	27/27.8	14/31.1	3/23.0	1/1.3	6/9.0	73/14.7	11.7–18.2
*Toxascaris leonina*	4/2.1	1/1.0	2/4.4	0/0.0	0/0.0	1/1.5	8/1.6	0.7–3.2
*Trichuris vulpis*	11/5.7	9/9.3	6/13.3	0/0.0	0/0.0	5/7.5	31/6.3	4.3–8.8
*Capillaria* spp.	4/2.1	0/0.0	2/4.4	0/0.0	0/0.0	1/1.5	7/1.4	0.6–2.9
*Ancylostoma/Uncinaria* spp.*	9/4.6	15/15.5	16/35.5	0/0.0	1/1.3	0/0.0	41/8.3	6.2–11.4
Taeniid species	8/4.1	2/2.1	6/13.3	1/7.8	0/0.0	3/4.5	20/4.0	2.5–6.2
*Echinococcus multilocularis* *	3/1.5	1/1.0	3/6.6	1/7.8	0/0.0	3/4.5	11/2.2	1.1–3.9
*Echinococcus canadensis* *	0/0.0	0/0.0	0/0.0	0/0.0	0/0.0	0/0.0	0/0.0	0.0–0.6
*Echinococcus granulosus s.s* *	0/0.0	0/0.0	0/0.0	0/0.0	0/0.0	0/0.0	0/0.0	0.0–0.6
*Taenia hydatigena*	2/1.0	0/0.0	0/0.0	0/0.0	0/0.0	0/0.0	2/0.4	0.1–1.2
*Taenia* spp.	3/1.5	1/1.0	3/6.6	0/0.0	0/0.0	0/0.0	7/1.4	0.6–2.9
*Dipylidium caninum* *	0/0.0	0/0.0	0/0.0	0/0.0	0/0.0	0/0.0	0/0.0	0/0.0
Totally Infected ** 95% CI	46/23.7 17.5–29.8	38/39.2 29.4–49.6	30/66.7 51.1–80.0	3/23.1 5.0–53.8	2/2.5 0.3–8.9	15/22.4 13.1–34.2	134/27.1 23.2–31.2	

*n*—number of examined; N—number of positive; %—prevalence; 95% CI—95% confidence interval; * zoonotic species; ** some animals suffered from mixed infection.

**Table 2:** Occurrence of mixed infections in dogs.

Occurrence of Mixed Infection	Pet Dogs *n* = 194 (N/%)	Shelter Dogs *n* = 97 (N/%)	Dogs from Segregated Roma Settlements *n* = 45 (N/%)	Guard Dogs *n* = 13 (N/%)	Working Dogs *n* = 79 (N/%)	Hunting Dogs *n* = 67 (N/%)	Total	
							*n* = 495 (N/%)	95% CI
One helminth species	36/18.5	29/29.9	19/42.2	2/15.4	2/2.5	14/20.9	102/20.6	17.1–24.4
Two helminth species	8/4.1	6/6.2	7/15.6	1/7.7	0/0.0	1/1.5	23/4.7	2.9–6.9
Three helminth species	2/1.0	3/3.1	4/8.9	0/0.0	0/0.0	0/0.0	9/1.8	0.83–3.4

*n*—number of examined; N—number of positive; %—prevalence; 95% CI—95% confidence interval.

## Data Availability

Not applicable.
